# Supplementary material for: Artificial intelligence in pancreatic intraductal papillary mucinous neoplasm imaging: A systematic review
Source: PLOS Digit Health. 2025 Jul 23;4(7):e0000920. doi: 10.1371/journal.pdig.0000920 (PMC12286379; doi:10.1371/journal.pdig.0000920)
Supplement: S1 Table — (PDF) [file pdig.0000920.s001.pdf]

Supplementary Table 1: Data extraction template. The table presents a structured data extraction template used to extract the data from the included studies on AI in IPMN imaging.

**Parameter**	**Description**	**Example**
Year of Publication	The year in which the study was published	2022
Ground Truth Source	Source of ground truth for the prediction target	Suspected diagnosis based on radiological imaging
Prediction Targets	The outcomes that the proposed model aims to predict based on input data	Risk stratification of IPMN
Number of Centers	The total number of centers the data was collected from	2
Patient Selection Criteria	The criteria used to identify and include patients in the study.	Preoperative CT of patients with a confirmed diagnosis of IPMN. CT within 2 weeks of surgery.
Number of Patients the AI Model was Developed On	Number of patients included in the study, including all patients from the training, validation, and test sets.	139
Number of Patients With IPMN the AI Model was Developed On	Number of all the patients in training, validation, and test sets diagnosed with IPMN.	110
Number of Patients (Training Set)	The number of patients AI was initially trained on	100
Number of Patients (Test Set)	Number of patients AI was tested on	39
Validation Procedure	The method used to assess the performance and reliability of a model	5-fold cross-validation or independent test set
Data/Imaging Modality	The type or modality of data AI model was trained on	MRI
Methodology	The computational methods, algorithms, and frameworks used to develop the AI model	nnUnet
Performance Metrics	Measures to assess the performance and accuracy of the model in predicting the outcome	Sensitivity: 95.7%, specificity: 92.6%, accuracy: 94.0%
Stage of Clinical Translation	Internal validation, external validation, prospective clinical evaluation, or FDA approval	External validation
