# Supplementary material for: Artificial intelligence in pancreatic intraductal papillary mucinous neoplasm imaging: A systematic review
Source: PLOS Digit Health. 2025 Jul 23;4(7):e0000920. doi: 10.1371/journal.pdig.0000920 (PMC12286379; doi:10.1371/journal.pdig.0000920)
Supplement: S2 Table — (PDF) [file pdig.0000920.s002.pdf]

Supplementary Table 2: Data extracted from the included publications on AI in IPMN imaging. Abbreviations used in the table: MPD: Main Pancreatic Duct; MCN: Mucinous Cystic Neoplasm; SCA: Serous Cystadenoma; SPN: Solid Pseudopapillary Neoplasm; SCN: Serous Cystic Neoplasm; SPT: Solid Pseudopapillary Tumor; MCA: Mucinous Cystadenoma; EUS-FNA: Endoscopic Ultrasound Fine Needle Aspiration; ICD: International Classification of Diseases; ND: Not Documented; CNN: Convolutional Neural Network; SVM: Support Vector Machine; GAN: Generative Adversarial Network; RF: Random Forest; ROI: Region of Interest; ViT: Vision Transformer; ANN: Artificial Neural Network; ML: Machine Learning; NB: Naive Bayes; KNN: K-Nearest Neighbor; LDA: Linear Discriminant Analysis; DL: Deep Learning, MRCP: Magnetic Resonance Cholangiopancreatography. Performance Metrics: DLM: Deep Learning Model; RM: Radiomics Model; Sen: Sensitivity; Spec: Specificity; Acc: Accuracy; Pre: Precision; Rec: Recall; AUC/AUROC: Area Under Receiver Operating Characteristic Curve; AUPR: Area Under Precision-Recall Curve; FPR: False Positive Rate; DSC: Dice Similarity Coefficient/Dice Score.

Author and Year of Publication	Prediction Targets	Ground Truth Source	Number of Centers	Patient Selection Criteria	Total Number of Patients AI was Developed on	Number of Patients with IPMN AI was Developed on	Number of Patients (Training Set)	Number of Patients (Testing Set)	Validation Procedure (cross validation vs. test set)	Imaging Modality	Methodology	Performance Metrics	Stage of Clinical Translation	Data Availability	Comments
**Abel et al. 2021[36]**	Detection of pancreatic cystic lesions (PCL)	Radiologically suspected	1	Inclusion: Portal venous CT scan, slice thickness of 1-1.5 mm, PCL described in the radiology report.<br>Exclusion: Report describing a pancreatic tumor, patient with pancreatitis, pseudocysts, and image artifacts.	221	173	221	ND	5-fold cross-validation	CT	nnU-Net for pancreas, cyst, and MPD segmentation; cyst detection via connected component analysis	Mean Sen: 78.8 ± 0.1%<br>Sen for different cyst volumes: 10-50: 40.1%; >50-200: 65.5%; >200-600: 75.9%; >600: 91.9%	Internal validation	All data openly available: https://zenodo.org/records/4621057	47 patients were selected randomly from training set to validate the model.
**AbiNader et al. 2023[37]**	Detection of pancreatic lesions, and prediction of MPD dilation	Pathological diagnosis or C25 ICD-10	9	Preoperative portal venous CT scan before any treatment, slice thickness ≤3mm, patients with confirmed pancreatic neoplasm.	2890	99	2134	756	5-fold cross-validation and test set	CT	3D nnUNet for segmentation of pancreas, lesions, and MPD; two logistic regression models to predict lesion presence and MPD dilatation	Segmentation: DSC 0.91 ± 0.06 (Pancreas); 0.69 ± 0.34 (Lesions); 0.58 ± 0.37 (MPD)<br>Lesion detection: AUC 0.98; Sen 94%; Spec 95%<br>IPMN: AUC 0.98; Sen 96%; Spec 94%<br>MPD dilation: AUC 0.97; Sen 94%; Spec 90%	External validation	-	Used public datasets as a part of validation set.
**Cao et al. 2023[38]**	Differential diagnosis of pancreatic lesions: PDAC and 7 non-PDAC lesions; and IPMN subtypes: Main/Mixed-duct or branch-duct	Pathological diagnosis	10	Patients with pathologically confirmed pancreatic lesions.	9939	529	3208	6731	5-fold cross-validation & test set	CT	Low resolution nnUNet to segment pancreas; multi-task CNN using full resolution nnUNet for lesion segmentation and detection; dual-path memory transformer for differential diagnosis of lesion subtypes; binary IPMN subtype classification in a cascaded fashion.	Internal test set:<br>Lesion detection: AUROC 0.996; Sen 94.9%; Spec 100%<br>PDAC identification: AUROC 0.987; Sen 92.6%; Spec 97.3%<br>Differential diagnosis: Acc 79.6%<br>IPMN subtype: AUC 0.944; Sen 94.1%; Spec 80.6%<br>External test set:<br>Lesion detection: AUROC 0.984; Sen 93.3%; Spec 98.8%<br>PDAC identification: Sen 90.1%; Spec 95.7%<br>Differential diagnosis: Acc 81.4%<br>IPMN subtype: AUC 0.915; Sen 89%; Spec 81.1%	Prospective clinical trial	Sample data available: panda.medofmind.com	
**Chu et al. 2022[39]**	Classification of Pancreatic Cystic Neoplasms (PCN)	Pathological diagnosis	1	Patients who underwent surgical resection for pancreatic cysts.	214	64	214	ND	4-fold cross-validation	CT	Random forest trained on extracted radiomic features for cyst-type classification.	Radiologist Prediction:<br>IPMN: Sen 90.6%; Spec 87.3%; Acc 88.3%; AUC 0.889<br>Overall: Sen 87.3%; Spec 90.6%; Acc 88.3%; AUC 0.895<br>Radiomics:<br>IPMN: Sen 93.8%; Spec 94.7%; Acc 94.4%; AUC 0.942<br>Overall: Sen 94.7%; Spec 93.8%; Acc 94.4%; AUC 0.940	Internal validation	-	20 patients were randomly selected from training set to evaluate the performance of model against radiologist.

**Corral et al. 2019[40]**	IPMN risk stratification: Normal, low-grade, and high-grade IPMN.	Pathological diagnosis	1	Patients diagnosed with IPMN, underwent surgery and had preoperative MRI.	139	108	139	ND	10-fold cross-validation	MRI	Minimum and maximum intensity projections computed from T1 and T2 images, fed into a pre-trained CNN to get feature representation; canonical correlation analysis for feature fusion; SVM for lesion classification.	DLM: AUC 0.78<br>To detect high-grade dysplasia or cancer: Sen 75%; Spec 78%<br>Fukuoka: AUC 0.77<br>To detect high-grade dysplasia or cancer: Sen 62%; Spec 77%<br>AGA: AUC 0.77<br>To detect high-grade dysplasia or cancer: Sen 25%; Spec 96%	Internal validation	-
**Dmitriev et al. 2021[41]**	Classification of PCLs: IPMN, MCN, SCA, SPN	Pathological diagnosis	ND	ND	194	122	134	60	Test set	CT	Probabilistic random forest to classify predefined features; CNN for analyzing radiological features; Bayesian combination to generate final probabilities for the lesion types.	Acc 91.7%	Internal validation	-
**Gao & Wang 2020[42]**	Classification of pancreatic diseases and cysts.	Pathological diagnosis	2	Preoperative Dynamic contrast-enhanced MRI exams for pancreatic lesions, post-operative pathological diagnoses, no artifacts, solitary lesions, and diameter of lesion ≥5 mm on the axial sections.	504	53	398	106	Test set	MRI	Trained GANs for data augmentation; transfer learning to train InceptionV4 to predict patch-level results; patient-level analysis using plurality voting to predict class from patch results.	Patch Level:<br>Internal: Mean Acc 71.56%; AUC 0.9204<br>External: Mean Acc 79.46%; AUC 0.9451<br>Patient Level:<br>Internal: Mean Acc 70%; AUC 0.8250<br>External: Mean Acc 76.79%; AUC 0.8646<br>Patient Level Human Reader:<br>Internal: Mean Acc 82%; AUC 0.8950<br>External: Mean Acc 83.93%; AUC 0.9063	External validation	-
**Hussein et al. 2018[43]**	IPMN risk stratification: Normal, low-grade, and high-grade IPMN.	Pathological diagnosis	ND	ND	139	108	139	ND	10-fold cross-validation	MRI	Minimum and maximum intensity projections computed from T1 and T2 images, fed into a pre-trained CNN to get feature representation; canonical correlation analysis for feature fusion; SVM for lesion classification.	Normal vs IPMN:<br>T1-weighted: Acc 84.23%; Sen 89.16%; Spec 55%<br>T2-weighted: Acc 61.04%; Sen 59.59%; Spec 57.67%<br>Concat. of T1 and T2: Acc 82.09%; Sen 88.49%; Spec 49.33%<br>Proposed: Acc 82.80%; Sen 83.55%; Spec 81.67%<br>Normal vs Low-Grade vs High-Grade IPMN<br>T1-weighted: Acc 58.3%<br>T2-weighted: Acc 45.93%<br>Concat. of T1 and T2: Acc 56.81%<br>Proposed: Acc 64.67%	Internal validation	-

**Hussein et al. 2019[44]**	Detection of IPMN	Labeled by radiologists	ND	ND	171	133	171	ND	10-fold cross-validation	MRI	Unsupervised Learning: Computing image features using GIST descriptors, K-mean clustering to obtain label proportions, and classification using $\propto$SVM. Compared with CNN extracted features (VGG-fc7/8) with and without ReLU non-linearity.<br>Supervised Learning: Trained RF and SVM on GIST, and VGGfc7/8 features.	Unsupervised IPMN Classification:<br>GIST features: Acc 58.04%; Sen 58.61%; Spec 41.67%<br>VGGfc7: Acc 49.12%<br>VGGfc7 with ReLu: Acc 51.47%<br>VGGfc8: Acc 50.36%<br>VGGfc8 with ReLu: Acc 50.92%<br>Supervised IPMN Classification:<br>GIST+SVM: Acc 76.05%; Sen 83.65%; Spec 52.67%<br>GIST+RF: Acc 81.9%; Sen 93.69%; Spec 43.0%<br>VGGfc7+SVM: Acc 84.18%; Sen 96.91%; Spec 44.83%<br>VGGfc7+RF: Acc 81.96%; Sen 94.61%; Spec 82.83%<br>VGGfc8+SVM: Acc 84.22%; Sen 97.2%; Spec 46.5%<br>VGGfc8+RF: Acc 80.82%; Sen 93.4%; Spec 45.67%	Internal validation	-
**Kuwahara et al. 2019[45]**	IPMN risk stratification: Benign vs malignant	Pathological diagnosis	1	Underwent EUS before pancreatic resection; pathologically confirmed IPMN after surgery; digital images.	50	50	50	ND	10-fold cross-validation	EUS	ResNet50	Threshold 0.49: AUROC 0.91; Sen 81.5%; Spec 90.1%; Acc 86.2%<br>Threshold 0.41: AUROC 0.98; Sen 95.7%; Spec 92.6%; Acc 94.0%<br>Human Diagnosis: Sen 0.957; Spec 0.222; Acc 0.560	Internal validation	-
**LaLonde et al. 2019[46]**	IPMN risk stratification: Normal, low-grade, and high-grade IPMN	Pathological diagnosis	ND	Patients who underwent surgical resection for IPMN	139	110	139	ND	10-fold cross-validation	MRI	Inflated neural networks based on InceptionV3 and DenseNet121, used early and intermediate fusion.	InceptINN Whole MRI:<br>Early Fusion: Pre 69.44%; Rec 66.35%; Acc 73.33%<br>Intermediate Fusion: Pre 66.67%; Rec 59.68%; Acc 66.67%<br>InceptINN Pancreas-ROI:<br>Early Fusion: Pre 79.29%; Rec 80.48%; Acc 78.57%<br>Intermediate Fusion: Pre 70.77%; Rec 69.66%; Acc 75%<br>DenseINN Pancreas-ROI:<br>Early Fusion: Pre 73.08%; Rec 73.08%; Acc 75%<br>Intermediate Fusion: Pre 88.10%; Rec 75.21%; Acc 82.14%	Internal validation	-
**Li et al. 2019[47]**	Classification of PCLs: IPMN, MCN, SCN, SPT	Pathological diagnosis	ND	Patients suspected of malignant cysts; no reported pancreatic disease before.	206	64	206	ND	10-fold cross-validation	CT	Dense-Net	Overall Acc: 72.8%<br>IPMN Acc: 81.25%	Internal validation	-

**Liang et al. 2022[48]**	Classification of PCLs: MCA vs IPMN	Pathological diagnosis	1	Inclusion: Patient who underwent resection; diagnosis of SCA, MCA, or IPMN; preoperative CT.<br>Exclusion: Poor image quality; incomplete CT or pathology report	94	39	94	ND	5-fold cross-validation	CT	IPMN vs MCA: radiomic model using SVM and a fused model using radiomic score, radiological features, and clinical features using logistic regression.	RM: AUC 0.900; Acc 90.3%; Sen 87%; Spec 94.9%<br>Fused Model: AUC 0.973; Acc 92.2%; Sen 86.3%; Spec 100%	Internal validation	Available upon request.	
**Mazor et al. 2023[49]**	Segmentation of pancreatic cysts	Ground truth segmentation by radiologists	1	Patients with pancreatic cysts undergoing IPMN follow-up.	158	158	118	23	Test and validation set	MRI	3D UNet to compute pancreas ROI in axial TSE; transfer pancreas ROI to MRCP. 3D Unet for detection and segmentation of pancreatic cysts in MRCP with pancreas ROI. Used hard negative patch mining to overcome the class imbalance.	For cysts > 10 mm:<br>Detection: Pre 0.95 ± 0.16; Rec 0.99 ± 0.05; F1 0.96 ± 0.11<br>Segmentation: DSC 0.81 ± 0.11<br>For cysts > 5 mm:<br>Detection: Pre 0.75 ± 0.26; Rec 0.80 ± 0.19; F1 0.75 ± 0.20<br>Segmentation: DSC 0.80 ± 0.08<br>All cysts:<br>Detection: Pre 0.61 ± 0.27; Rec 0.80 ± 0.22; F1 0.65 ± 0.23<br>Segmentation: DSC 0.80 ± 0.09	Internal validation	-	
**Park et al. 2023[50]**	Detection of solid and cystic pancreatic neoplasms	Pathological diagnosis	2	Inclusion: Patients who underwent resection for pancreatic neoplasms with preoperative CTs, and controls.<br>Exclusion: Tumor invisible in CT, interval between surgery and CT >30 days, prior treatment of pancreatic tumor, image artifacts, and biliopancreatic drainage tube.	2044	132	852	1192	5-fold cross-validation and test set	CT	3D nnUnet for segmentation of pancreas and pancreatic lesions; voting ensemble-based classifier.	Test Set 1:<br>DLM: AUC 0.91; Sen 90.2%; Spec 85.1%; Acc 86.2%<br>Radiologist 1: AUC 0.92; Sen 91.7%; Spec 93.2%; Acc 92.9%<br>Radiologist 2: AUC 0.95; Sen 95.5%; Spec 94.3%; Acc 94.5%<br>Test Set 2:<br>DLM: AUC 0.87; Sen 83.3%; Spec 82.7%; Acc 82.9%<br>Radiologist 1: AUC 0.95; Sen 94.7%; Spec 95.6%; Acc 95.4%<br>Radiologist 2: AUC 0.96; Sen 95.6%; Spec 96.2%; Acc 96.1%	External validation	-	
**Qu et al. 2023[51]**	Classification of PCLs prone to non-diagnostic EUS-FNA from those prone to conclusive EUS-FNA	Pathological diagnosis or medical records	1	Inclusion: Diagnosis of solid pancreatic cancer, contrast enhanced CT within 14 days before EUS-FNA.<br>Exclusion: Cystic lesions or needle size was not 22G.	221	8	147	74	Test set	CT	PyRadiomics to extract radiomic features, a deep neural network for classification.	Sen 78.3%; Spec 50%; Acc 67.6%; AUC 0.745<br>Extended Application: Sen 78.3%; Spec 42.9%; Acc 64.9%; AUC 0.624	Internal validation	-	IPMN patients were only included to test for extended application not in training set.
**Salanitri et al. 2022[52]**	IPMN Risk stratification : Normal, low-grade, and high-grade IPMN	Pathological diagnosis	1	Patients with IPMN or normal control.	139	108	139	ND	10-fold cross-validation	MRI	Fine-tuned pre-trained ViTs; early and late fusion of T1 and T2 weighted MRI	T1: Acc 0.53 ± 0.08; Pre 0.60 ± 0.11; Rec 0.58 ± 0.14<br>T2: Acc 0.64 ± 0.12; Pre 0.64 ± 0.13; Rec 0.63 ± 0.11<br>Early Fusion: Acc 0.70 ± 0.11; Pre 0.67 ± 0.19; Rec 0.64 ± 0.12<br>Late Fusion: Acc 0.60 ± 0.16; Pre 0.61 ± 0.13; Rec 0.59 ± 0.11	Internal validation	-	

**Schulz et al. 2023[53]**	IPMN Risk stratification: Low-grade vs high-grade IPMN /invasive carcinoma	Pathological diagnosis	2	Inclusion: Patients who underwent pancreatectomy. Exclusion: IPMN as a secondary finding to pancreatic cancer or if there was no preoperative EUS	70	70	43	27	Test set	EUS	Transfer learning with Efficient Net B5 backbone	DLM: Acc 99.6%; Sen 100%; Spec 99.7%<br>2015 AGA: Acc 70.3%; Sen 25.0%; Spec 89.5%<br>2017 Revised Fukuoka: Acc 70.4%; Sen 87.5%; Spec 63.2%<br>2018 ACG: Acc 51.8%; Sen 100%; Spec 31.6%<br>2018 European: Acc 51.9%; Sen 87.5%; Spec 36.8%	External validation	-
**Shen et al. 2020[54]**	Classification of PCLs: SCA, MCN, IPMN	Pathological diagnosis	1	Contrast enhanced CT scan within 2 weeks before surgery, postoperative pathological diagnosis of SCA, MCN, or IPMN.<br>No hepatic pancreaticobiliary malignancies, image artifacts, or missing data.	164	48	115	49	Validation set	CT	Extraction of clinical and radiomic features; trained three machine learning models: SVM, RF, and ANN.	SVM: Acc 71.43%<br>RF: Acc 79.59%<br>ANN: Acc 71.43%	Internal validation	-
**Si et al. 2021[55]**	Detection of pancreatic tumors	Labeled by radiologists	2	Underwent preoperative abdominal contrast-enhanced CT.	666	23	319	347	Test set	CT	ResNet18 to detect pancreas; U-Net32 for segmentation of the pancreas; ResNet34 to detect tumors	Overall: AUC 0.871; F1 88.5%; Acc 82.7%; Sen 86.8%; Spec 69.5%<br>IPMN: Acc 100%	External validation	-
**Wang et al. 2022[56]**	Classification of PCLs: Benign (includes low-grade IPMN) or malignant (includes high-grade or invasive IPMN)	Pathological diagnosis	2	Inclusion: Reported PCLs on the CT. Exclusion: No pathological results within 3 months, incomplete clinical data, treatment before surgery, image artifacts.	363	65	266	102	10-fold cross-validation and test set	CT	Radiomics Model: Radiomic features were extracted; ML models (SVM, RF, AdaBoost, XGBoost, Bernoulli NB, KNN, LDA) were trained.<br>Deep Learning Model: Densely connected neural network	Internal test:<br>RM: AUC 0.879; Acc 0.750; Sen 0.545; Spec 0.900<br>DLM: AUC 0.933 Acc 0.904 Sen 1.00; Spec 0.833<br>Senior Radiologist: Acc 0.885; Sen 0.955; Spec 0.833<br>Junior Radiologist: Acc 0.750; Sen 1.000; Spec 0.567<br>Surgeon: Acc 0.769; Sen 0.864; Spec 0.700<br>External test:<br>RM: AUC 0.768; Acc 0.720; Sen 0.536; Spec 0.955<br>DLM: AUC 0.911 Acc 0.840 Sen 0.964; Spec 0.682<br>Senior Radiologist: Acc 0.920; Sen 0.857; Spec 1.000<br>Junior Radiologist: Acc 0.700; Sen 0.929; Spec 0.409<br>Surgeon: Acc 0.640; Sen 0.679; Spec 0.591	External validation	-

**Watson et al. 2021[57]**	Classification of PCNs: Benign (intermediate/low-grade IPMN/SCA) or malignant (adenocarcinoma/high-grade IPMN)	Pathological diagnosis	1	Inclusion: Diagnosis of IPMN, MCN, or SCN, pathology report available. Exclusion: Patients with pancreatic pseudocysts, no preoperative CT	27	15	18	9	5-fold cross-validation and test set	CT	LeNet architecture	DLM: Acc 8/9 ~ 88%<br>Fukuoka: Acc 6/9 ~ 66%	Internal validation	-	
**Yao et al. 2023[58]**	IPMN risk stratification: Normal, low-grade, and high-grade IPMN	Pathological diagnosis or radiographical evaluation	5	ND	246	176	197	49	5-fold cross-validation and test set	MRI	3D nnUNet for segmentation. Radiomics model: feature extraction from the ROI and clinical features, used XGBoost for classification. Deep learning model: ViT; fusion of weighted probabilities of DL and radiomic model.	Segmentation DSC: 70.11%<br>ViT w/o Radiomics: Acc 61.3%; AUC 71.9%; Pre 56.2%; Rec 56.6%<br>ViT w Radiomics: Acc 81.9%; AUC 89.3%; Pre 82.4%; Rec 82.7%	External validation	-	Data from centers merged into one dataset and randomly split into training and test sets.
**Yuan et al. 2023[59]**	Segmentation of medical images	Pathological diagnosis and ground truth segmentation by radiologists	ND	Consecutive patients contrast-enhanced CT imaging with tumor types confirmed by pathology.	661	ND	378	118	Test and validation set	CT	nnUNet with a transformer decoder	Outlier detection (pixel level): AUROC 82.52%; AUPR 55.60%; FPR95 46.19%<br>Outlier detection (case level): AUC 77.97%<br>Inlier Segmentation DSC: IPMN 46.92%; Mean 41.77%	Internal validation	-	
**Zhang et al. 2022[60]**	Classification of PCNs: Benign or malignant & four-class classification: SCN, MCN, IPMN, SPN	Pathological diagnosis	1	Inclusion: Underwent pancreatectomy for PCN; diagnosed with SCN, MCN, IPMN, or SPN; CT scan available 1 month before surgery. Exclusion: Insufficient medical records; image artifacts; unable to make a clinical diagnosis.	263	66	263	ND	5-fold cross-validation	CT	CNN for feature extraction and graph neural network for classification	Binary classification: Acc 88.92%; Sen 89.93%; Spec 88.59%; Pre 72.43%; F1 80.24%<br>4-class classification: Acc 74.32%; F1 IPMN 74.26%<br>Different ROIs:<br>Binary classification: Pancreatic region: Acc 81.37%; only neoplasm 88.92%<br>4-class classification: Pancreatic region: 74.49%, Only neoplasm 74.32%	Internal validation	-	
