# Supplementary material for: Artificial intelligence in pancreatic intraductal papillary mucinous neoplasm imaging: A systematic review
Source: PLOS Digit Health. 2025 Jul 23;4(7):e0000920. doi: 10.1371/journal.pdig.0000920 (PMC12286379; doi:10.1371/journal.pdig.0000920)
Supplement: S3 Table — (PDF) [file pdig.0000920.s003.pdf]

Supplementary Table 3: Demographic characteristics of the included studies. * indicates that the estimate is based on training data only. † indicates that the mean or median was pooled across all pathologies. ND: Not Documented.

Study ID	Country	Age	Sex Ratio
**Abel 2021[36]**	Switzerland	Mean: 72.9 ± 12.7	1.66
**AbiNader 2023[37]**	France	Median: 64 [56,73] *	1.22*
**Cao 2023[38]**	China	Median: 58 †*	0.77*
**Chu 2022[39]**	USA	Mean: 54.8 ± 17.0	2.1
**Corral 2019[40]**	USA	Mean: 65.3 ± 11.9	1.4
**Dmitriev 2021[41]**	USA	ND	2.12*
**Gao 2020[42]**	China	Mean: 59.14 ± 12.07 *	0.85*
**Hussein 2018[43]**	USA	ND	ND
**Hussein 2019[44]**	USA	ND	ND
**Kuwahara 2019[45]**	Japan	Median: 66 [18-81]	1
**LaLonde 2019[46]**	USA	ND	ND
**Li 2019[47]**	Germany, China, Switzerland	Mean: 53.4 ± 15.1	1.54
**Liang 2022[48]**	China	Mean: 52.2 ± 14.2 †	2.36
**Mazor 2023[49]**	Israel	ND	ND
**Park 2023[50]**	Korea	Median: 60 [19-85] *	0.84*
**Qu 2023[51]**	China	Mean: 59.27 ± 9.03 †*	0.47*
**Salanitri 2022[52]**	USA	Mean: 65.3 ± 11.9	1.4
**Schulz 2023[53]**	Germany	Mean: 70.84 †*	1.34*
**Shen 2020[54]**	China	Median: 57 [20–79] *	2.28*
**Si 2021[55]**	China	Mean: 63.3*	0.51*
**Wang 2022[56]**	China	Mean: 47.75 †*	1.48*
**Watson 2021[57]**	USA	Median: 69 [57.8-73]	0.73
**Yao 2023[58]**	USA	ND	ND
**Yuan 2023[59]**	China	ND	ND
**Zhang 2022[60]**	China	ND	ND
