# Supplementary material for: Artificial intelligence in pancreatic intraductal papillary mucinous neoplasm imaging: A systematic review
Source: PLOS Digit Health. 2025 Jul 23;4(7):e0000920. doi: 10.1371/journal.pdig.0000920 (PMC12286379; doi:10.1371/journal.pdig.0000920)
Supplement: S4 Table — (PDF) [file pdig.0000920.s004.pdf]

Supplementary Table 4: Risk of bias assessment using PROBAST Tool

Study ID	Participants	Predictor	Outcome	Analysis	Overall
**Abel 2021[36]**	Low	Low	Unclear	Low	High
**AbiNader 2023[37]**	Low	Low	Unclear	Low	Unclear
**Cao 2023[38]**	Low	Low	Low	Low	Low
**Chu 2022[39]**	Unclear	Low	Low	Unclear	High
**Corral 2019[40]**	Low	Low	Low	Unclear	High
**Dmitriev 2021[41]**	High	Low	Low	High	High
**Gao 2020[42]**	Low	Low	Low	Low	Low
**Hussein 2018[43]**	High	Low	Low	Unclear	High
**Hussein 2019[44]**	High	Low	High	Low	High
**Kuwahara 2019[45]**	Low	Low	Low	Unclear	High
**LaLonde 2019[46]**	High	Low	Low	Unclear	High
**Li 2019[47]**	Unclear	Low	Low	Unclear	High
**Liang 2022[48]**	Low	Low	Low	Low	High
**Mazor 2023[49]**	Unclear	Low	Low	Low	Unclear
**Park 2023[50]**	Low	Low	Low	Low	Low
**Qu 2023[51]**	Low	Low	Unclear	High	High
**Salanitri 2022[52]**	Unclear	Low	Low	Low	High
**Schulz 2023[53]**	Low	Low	Low	Low	Low
**Shen 2020[54]**	Low	Low	Low	Unclear	Unclear
**Si 2021[55]**	Unclear	Low	Unclear	Low	Unclear
**Wang 2022[56]**	Low	Low	Low	Low	Low
**Watson 2021[57]**	Low	Low	Low	High	High
**Yao 2023[58]**	Unclear	Low	Unclear	Low	Unclear
**Yuan 2023[59]**	Low	Low	Low	Low	Low
**Zhang 2022[60]**	Low	Low	Low	Low	High
