# Supplementary figures and images for: Artificial intelligence in pancreatic intraductal papillary mucinous neoplasm imaging: A systematic review
Source: PLOS Digit Health. 2025 Jul 23;4(7):e0000920. doi: 10.1371/journal.pdig.0000920 (PMC12286379; doi:10.1371/journal.pdig.0000920)

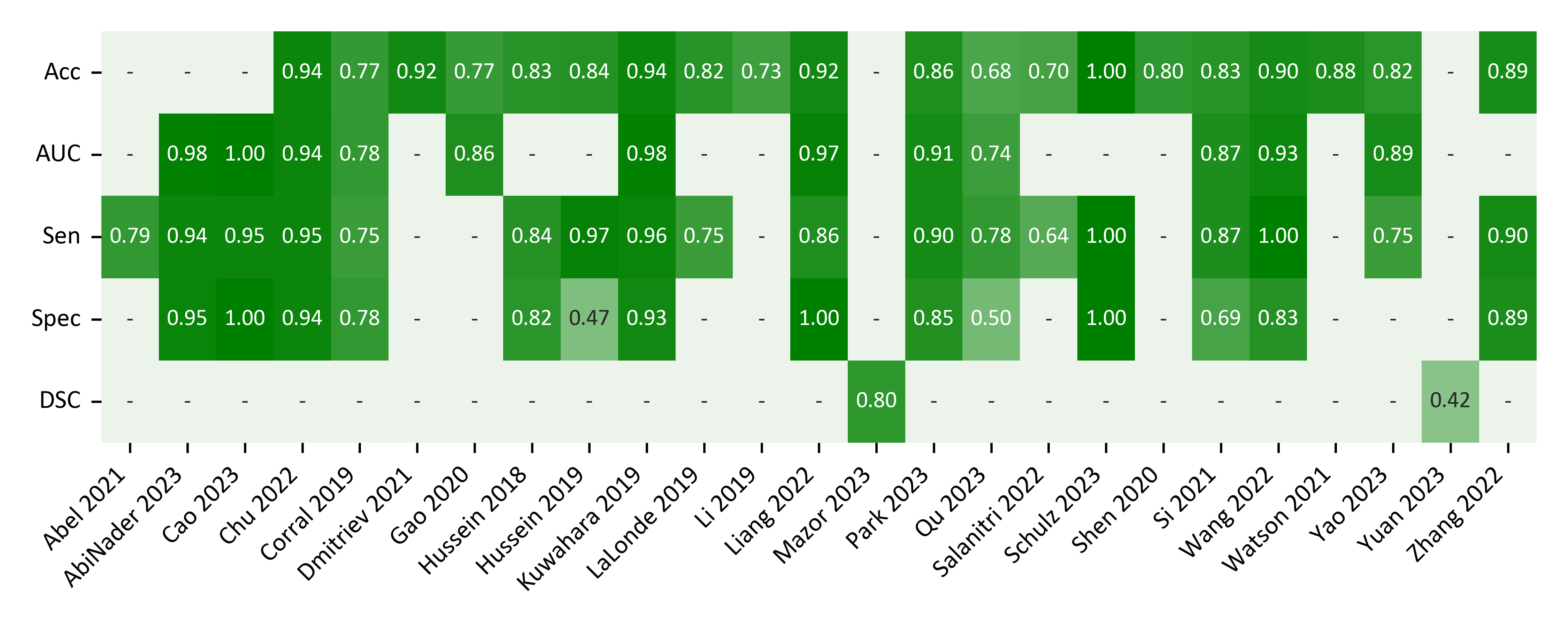

Supplement: S1 Fig — For detection, differential diagnosis, and risk stratification accuracy (Acc), area under the receiver operating curve (AUC), sensitivity (Sen), and specificity (Spec) are presented. For segmentation, the DSC is presented. — indicates that the metric was not reported. (PNG) [file pdig.0000920.s005.png]
