# Supplementary material for: Artificial intelligence in pancreatic intraductal papillary mucinous neoplasm imaging: A systematic review
Source: PLOS Digit Health. 2025 Jul 23;4(7):e0000920. doi: 10.1371/journal.pdig.0000920 (PMC12286379; doi:10.1371/journal.pdig.0000920)
Supplement: Attachment — Submitted filename: Point-by-Point-Response_AI-in-IPMN-Imaging-Systematic-Review.pdf [file pdig.0000920.s010.pdf]

Point-by-Point Response

Editorial Office Comments

Thank you for submitting your manuscript to PLOS Digital Health. After careful consideration, we feel that it has merit but does not fully meet PLOS Digital Health's publication criteria as it currently stands. Therefore, we invite you to submit a revised version of the manuscript that addresses the points raised during the review process.

Thank you. We have carefully revised our manuscript and have integrated several substantive changes based on the reviewer comments:

- *We have added a new Supplementary Table 3, which details the demographic characteristics of the included studies, including study location, average patient age, and female-to-male ratio.*
- *The Discussion section has been substantially expanded to address key reporting, regulatory, and ethical challenges related to the application of Artificial Intelligence (AI) in the clinical management of Intraductal Papillary Mucinous Neoplasms (IPMN).*
- *To ensure the review remains up-to-date, we conducted an additional scoping search covering studies published between February 1, 2024, and May 31, 2025. The seven identified studies have been incorporated in the revised discussion to reflect the most current advances in the field.*

Reviewer #1

The systematic review is written in an intelligible fashion and concisely provides the research rationale and overall aim of the study. As well highlighted by the research, there is a great need to improve the diagnosis of pancreatic cancer while also avoiding surgical overtreatment. The paper describes novel applications of AI in IPMN imaging.

> *Thank you for your positive feedback and helpful comments.*

In terms of the revisions for this paper, I recommend minor revisions. Firstly, in the introduction of the paper, diagnostic errors (late or misdiagnosis of a disease) have been described, but the key term is not mentioned. Therefore, I would include this term and its definition, along with key statistics reported within the literature. For example, the number of diagnostic errors related to pancreatic cancer seen annually. **[R1.1]**

> *We thank Reviewer #1 for highlighting this. In response, we have revised the introduction to explicitly include the term "late diagnosis" and contextualize it with relevant epidemiological data. Specifically, we incorporated findings from a recent study conducted at a tertiary care Veterans Affairs medical center in the United States, which reported that over one-third of pancreatic cancer cases are diagnosed at an advanced stage [1]. Additionally, we also included that only approximately 20% of patients present with surgically resectable disease [2], highlighting the significant clinical burden of delayed diagnosis. These additions emphasize the critical need for earlier diagnostic strategies in pancreatic cancer.*

With regards to the methods, the systematic review has demonstrated the use of systematic review protocols and tools. Moreover, the systematic review protocol has been submitted and registered with PROSPERO. Although individual studies have not been quality checked, the authors have explained the rationale for not carrying out individual assessments within the discussion section of this review. However, more details should be given as to why the established quality tools were not suitable for the types of studies included. **[R1.2]**

> *Thank you for pointing this out. We would like to clarify that all the studies included in our systematic review were assessed using the PROBAST risk of bias assessment tool [3], which is specifically developed for evaluating prediction model studies (Table 1, Supplementary Table 4). We have revised the discussion section to more clearly explain our rationale for selecting a flexible framework like PROBAST over tools like QUADAS (which are designed specifically for diagnostic accuracy studies) [4]. We also have explicitly stated that a risk of bias assessment was performed at the individual study level.*

Please mention within the 'Study Characteristics' section the type of studies included, such as case-control or cohort, retrospective or prospective. The authors can also include study characteristics such as the female-to-male ratio of participants, participants' age/age range, and the country where the study took place, if mentioned within the literature. **[R1.3]**

> *Thank you for this helpful suggestion. We have revised the Study Characteristics section to note that all included studies were retrospective in nature. Additionally, we have*

included a new supplementary table (Supplementary Table 3) that details key study-level characteristics, including participant age, sex distribution, and country of origin, where reported. However, we note that some studies did not provide detailed demographic information, and among those that did, reporting formats varied (e.g., mean vs. median age). We have clarified this in the legend of the supplementary table.

In Table 1, the authors have identified that several of the papers demonstrated a high risk of bias. Please highlight that these papers were approached with caution or further explain the inclusion of such papers. **[R1.4]**

Thank you for this comment. We agree that studies identified as having a high risk of bias should be interpreted with caution. As such, we have revised the results section, mentioning the absolute number and proportion of high, low, and unclear risk of bias studies in our review. We have also revised the discussion section, highlighting that these studies were included for completeness but were interpreted carefully in light of their methodological limitations. This approach aligns with common practices in systematic reviews of prediction models [5,6], where excluding high-risk studies may introduce selection bias and limit comprehensiveness.

The authors have made apparent heterogeneity within the methodologies of the included studies. Please explain why this variation was seen? **[R1.5]**

We appreciate this important comment. The observed methodological heterogeneity across the included studies is largely due to the absence of a "benchmarking" or "lighthouse" study that establishes a single preferred approach for AI-based prediction in IPMN imaging. As a result, individual research groups have adopted varying modeling strategies, preprocessing techniques, and validation methods, contributing to the variability in methodologies. We have clarified this point in the revised discussion section.

Within the discussion section, the authors have suggested a lack of accuracy of studies with low participant numbers. It is important to consider the generalisability of these studies. **[R1.6]**

Thank you very much. We have revised the discussion to clarify that studies with small sample sizes may limit the generalizability of the findings to a broader population.

Although figures 1 - 3 are highly informative and aid in the understanding of the research, the quality/resolution of images must be improved prior to publication. **[R1.7]**

Thank you. We have reviewed all figures and ensured they comply with the guidelines of PLOS Digital Health. All figures have been re-uploaded at a resolution of 600 dpi.

A potential error was noticed within S3 Table. The Study by Zhang 2022 received low bias ratings for participants, predictors, outcome, and analysis but received an overall bias rating of high. **[R1.8]**

Thank you for bringing this to our attention. As noted in the PROBAST guidelines [3], "If a prediction model was developed without any external validation, and it was rated as low

risk of bias for all domains, consider downgrading to high risk of bias. Such a model can only be considered as low risk of bias if the development was based on a very large data set and included some form of internal validation." While the study by Zhang et al. (2022) received low risk ratings across all individual domains, it did not include external validation, and the dataset comprised only 263 patients, which is not a very large cohort. Therefore, the overall risk of bias was appropriately downgraded to high.

Overall, this paper was intriguing and highlighted a novel application for AI of IPMN for the purpose of pancreatic cancer diagnosis.

Thank you once again for your thoughtful and constructive feedback.

Reviewer #2

The systematic review provides a solid foundation for understanding the role of AI in IPMN imaging. It is comprehensive in its examination of imaging modalities, prediction targets, and publication trends.

> *We sincerely thank Reviewer #2 for their positive feedback and thoughtful review. We are pleased that the scope and depth of our systematic analysis were well received. We truly appreciate your time and constructive evaluation.*

Reviewer #3:

The paper entitled "Artificial Intelligence in Pancreatic Intraductal Papillary Mucinous Neoplasm Imaging: A Systematic Review" provides a systematic review that addresses a timely and relevant topic by evaluating the current landscape of AI applications in imaging for pancreatic intraductal papillary mucinous neoplasms (IPMNs). The paper is well-structured, comprehensive, and generally well-written. The authors rigorously applied PRISMA and AMSTAR guidelines and thoughtfully included a risk of bias assessment (PROBAST), which strengthens the methodological validity of the review. The inclusion of multiple imaging modalities (CT, MRI, EUS) and stratification of AI use cases (detection, segmentation, differential diagnosis, risk stratification) allows for a nuanced understanding of the field. The paper effectively identifies key research gaps, including small, single-center studies and limited clinical translation, while emphasizing the need for multimodal data and multicenter collaboration to advance AI in this field.

> *We sincerely thank Reviewer #3 for the positive feedback and helpful and thorough review. We are pleased that the methodological rigor and comprehensive scope of our systematic review were well received. Following your recommendations, we have included substantial changes in the revised version of the manuscript.*

Although the manuscript focuses on AI applications in IPMN imaging, many included studies do not centre exclusively on IPMN. Please clarify and justify the inclusion of studies where IPMN is a subset within broader pancreatic cyst or lesion cohorts. Consider stratifying the results more distinctly to delineate findings specific to IPMN. **[R3.1]**

> *Thank you for this important comment. We agree that not all included studies focused exclusively on IPMN. However, we included them based on our predefined inclusion criteria, which allowed studies analyzing IPMN or broader cystic lesion cohorts with relevance to IPMN. Many of these studies included mixed pancreatic cyst populations, and all included studies developed models applicable to IPMN.*
>
> *We have revised the Methods: Study Selection section to clarify this point.*
>
> > *The inclusion criteria were: (i) analysis of IPMN (and synonyms: mucinous cyst, mucinous cystic lesion) imaging, including the analysis of broader pancreatic cystic lesions if the reported findings were relevant to IPMN or if data from patients with IPMN were used.*
>
> *As already noted in the manuscript, Table 1 specifies the proportion of IPMN cases within each study cohort. Additionally, in the Discussion section (paragraph 2), we have highlighted the inclusion of broader pancreatic cystic lesion cohorts.*

While the manuscript reports various performance metrics (AUC, accuracy, etc.), more commentary on statistical rigor (e.g., confidence intervals, statistical significance, inter-rater agreement during data extraction) would enhance transparency. Were metrics statistically compared across models or studies? **[R3.2]**

Thank you for this valuable suggestion. We have reported all the available performance metrics in Figure 3C, Table 1, and Supplementary Table 2 extracted from the included studies. However, due to inconsistent reporting across studies—many of which did not report confidence intervals, p-values, or measures of variability—it was not feasible to perform a formal statistical comparison of model performance.

This limitation reflects a well-recognized issue in the field of AI in healthcare [7], as highlighted in these systematic reviews and commentaries [8,9], which have similarly noted incomplete and non-standardized reporting of model evaluation metrics. The Discussion section acknowledges this limitation and reinforces the need for improved reporting standards in future studies.

The discussion would benefit from deeper analysis of specific regulatory, technical, or economic barriers that may explain the lack of FDA-approved AI tools for IPMN, in contrast with other cancers such as breast or lung. **[R3.3]**

Thank you very much for bringing up this highly relevant point. We fully agree and have now expanded the Discussion section to address potential regulatory, technical, and clinical barriers that may underlie the lack of FDA-approved AI tools for IPMN. In this context, we also highlight the recent FDA Breakthrough Device designation granted to PANDA—a deep learning model developed for early detection of pancreatic ductal adenocarcinoma—from a study included in our review [10]. This example underscores both the potential and the current limitations in translating AI tools for pancreatic cancer into clinical practice.

Although the paper states that data is available in the manuscript and supplementary files, it's unclear if raw data or code used in the review (e.g., data extraction sheets, PROBAST assessments) are available for reproducibility. Consider depositing these in a public repository. **[R3.4]**

Thank you for this helpful feedback. The full data extraction (Supplementary Tables 2 and 3) and the PROBAST risk of bias assessments (Supplementary Table 4) are provided in the supplementary materials to ensure transparency and reproducibility. As per the journal's guidelines, all relevant data are mentioned in the Supporting Information section following the References.

The manuscript is generally intelligible and written in standard academic English. However, a few grammatical issues and overly complex sentence constructions in the introduction and discussion sections could be revised for better readability. For example, the sentence beginning "Given the substantial risks associated with pancreatic surgery..." is quite dense and could be simplified. **[R3.5]**

Thank you for this constructive comment. We have carefully reviewed the manuscript, particularly the Introduction and Discussion sections, and have revised several sentences to improve readability. The sentence highlighted by the reviewer has also been rephrased for better flow.

Consider including a summary table or heatmap of PROBAST scores across studies in the main text to better visualize common sources of bias across domains (participants, predictors, outcomes, analysis). **[R3.6]**

Thank you. A detailed summary of PROBAST scores across all studies has already been provided in Supplementary Table 4. This allows readers to explore the full risk of bias assessments in depth while maintaining flow in the main text. Again, we have ensured that this is clearly referenced in the manuscript for ease of access.

While heterogeneity precludes a formal meta-analysis, the inclusion of a weighted average performance score (e.g., average AUC or accuracy across use cases) or a summary figure of model performance could help readers synthesize the findings quantitatively. **[R3.7]**

Thank you for this suggestion. We agree that quantitative synthesis is helpful for better understanding. We have included a summary of model performance, visualizing available performance metric values across use cases (Figure 3C) and individual studies (Supplementary Figure 1). These figures were already included in the previous version of the manuscript and were designed to help readers synthesize and interpret performance trends across studies.

The discussion introduces potential applications of large language models (LLMs), such as GPT-4 and Med-PaLM 2, but lacks depth. A more critical exploration of how vision-language models could be integrated into IPMN workflows would add a forward-looking component. **[R3.8]**

We thank the reviewer for this comment. In response, we have substantially expanded the discussion to critically explore how vision-language models could be integrated into IPMN workflows. The revised paragraph now includes potential applications such as structured radiology report generation, multimodal risk stratification, and alignment with clinical guidelines using techniques like Retrieval-Augmented Generation. These additions are intended to provide a more forward-looking perspective for future research in this area.

While the review is focused on technical aspects, it may be worthwhile to briefly discuss ethical considerations in applying AI to high-stakes surgical decision-making, especially given the overtreatment risks in IPMN. **[R3.9]**

Thank you for this important suggestion. We agree that ethical considerations are critical when applying AI to high-stakes surgical decisions, particularly in the context of potential overtreatment in IPMN. We have now added a brief paragraph in the revised Discussion section addressing ethical concerns such as algorithmic bias, explainability, and the need for clinician oversight to ensure responsible deployment of AI tools in clinical settings.

Some terms and abbreviations (e.g., RM, ViT, DLM) are introduced without a clear definition in the text. Consider including a glossary or ensuring all are defined at first use. **[R3.10]**

Thank you for pointing this out. These abbreviations are used in Table 1 and are defined in full in the table legend for clarity. We have reviewed the manuscript to ensure consistent and clear use of terminology and abbreviations throughout.

Reviewer #4

The authors conducted a systematic review to assess the potential of AI-based methods in enhancing diagnosis and risk stratification for intraductal papillary mucinous neoplasm (IPMN). They categorized the selected studies based on prediction targets, imaging modality and data type, patient cohort size, and stage of clinical translation. A major limitation identified across studies was the scarcity of large, high-quality datasets, particularly multicenter and publicly available ones.

> *Thank you very much for your helpful feedback and suggestions.*

Considering the rapid development of AI in medical imaging, it would be beneficial for the authors to include studies published in 2024. This would ensure that the review remains current and does not miss recently developed models or advancements that might significantly impact the field by the time of publication. **[R4.1]**

> *We thank Reviewer #4 for this valuable suggestion and agree that consideration of studies published in 2024 would increase the actuality of the review. Therefore, we conducted an additional scoping search covering the period from February 1, 2024, to May 31, 2025, using the same search strategy and inclusion criteria as our initial one. This scoping search identified seven additional studies that met our criteria.*
>
> *These recent publications have been incorporated in the Discussion section, where we highlight several key developments, i.e., increased utilization of EUS-based AI models, early exploration of federated learning approaches for multi-institutional training, and modest but important progress toward external validation of existing algorithms. Additionally, we identified one study with a prospective validation and one that explored federated learning.*
>
> *We believe that the consideration of these studies in the Discussion strengthens our review by ensuring comprehensive coverage of the rapidly evolving landscape and provides readers with the most current understanding of AI applications for IPMN diagnosis and management. At the same time, these changes keep our review aligned with our pre-registered study protocol [11,12].*

While the paper clearly identifies major limitations such as the lack of high-quality, large-scale datasets and limited clinical translation, it would strengthen the manuscript if the authors could offer concrete suggestions or discuss potential strategies to overcome these challenges. **[R4.2]**

> *Thank you. We agree that highlighting strategies to overcome current limitations would strengthen the discussion. In response, we have revised the Discussion section to include specific suggestions for advancing the field, including the need for large, multicenter annotated datasets, public data sharing initiatives, standardized reporting practices, and clinically focused prospective validation. This lays out a practical roadmap for future work in this area.*

Reviewer #5

This systematic review provides a timely and comprehensive synthesis of the current state of AI applications in IPMN imaging. The manuscript is well-structured, adhering to PRISMA and AMSTAR guidelines, and addresses a clinically relevant gap in the specificity of IPMN management. The authors effectively highlight the potential of AI to mitigate overtreatment and improve diagnostic accuracy. However, several methodological and translational limitations warrant discussion to strengthen the manuscript.

Strengths
Scope and Rigor: The review encompasses 25 studies (2018–2023), offering a robust analysis of AI methodologies (e.g., CNNs, radiomics) and imaging modalities (CT, MRI, EUS). The stratification by prediction targets (detection, segmentation, diagnosis, risk stratification) is particularly insightful.
Clinical Relevance: The discussion on the limitations of Fukuoka/Kyoto guidelines and the role of AI in enhancing specificity is compelling. The emphasis on multicenter collaboration and multimodal data integration aligns with unmet clinical needs.
Transparency: Prospective registration (OSF, PROSPERO) and detailed risk-of-bias assessment (PROBAST) enhance reproducibility.

Thank you very much for your detailed feedback.

Limitations and Suggestions for Improvement

Heterogeneity of Studies: The wide variability in cohort sizes (e.g., 27 to 9,939 patients), imaging modalities, and performance metrics (e.g., AUC: 0.624–0.996) complicates cross-study comparisons. A meta-analysis might be unfeasible, but a qualitative synthesis of high-performing models (e.g., nnUNet for segmentation) could be expanded. Suggestion: Include a table summarizing top-performing models per use case with key metrics (e.g., sensitivity, specificity) and dataset characteristics. **[R5.1]**

We thank Reviewer #5 for their constructive suggestion. To address this point, we have added Supplementary Table 3, which summarizes key demographic characteristics of the included datasets. Table 1 and Supplementary Table 2 already provide detailed information on datasets, use cases, model types, and reported performance metrics (e.g., AUC, accuracy, sensitivity, specificity). Where available, we have noted the best-performing models in the Results section. Due to the heterogeneity of methodologies, a formal meta-analysis remains infeasible, but we believe these additions enhance the qualitative comparison across studies.

Data Availability and Bias: Most studies used single-centre, retrospective datasets with unclear selection criteria, raising concerns about generalizability. Only 32% of studies underwent external validation. Suggestion to discuss potential biases (e.g., selection, spectrum bias) and advocate for standardized reporting (e.g., STARD-AI, TRIPOD-AI) in future studies. **[R5.2]**

Thank you for this insightful comment. The revised Discussion section now explicitly addresses the limitation arising from single-center, retrospective data with unclear

inclusion criteria. We now advocate for the adoption of standardized reporting frameworks such as CLAIM (specifically designed for AI in medical imaging) to improve transparency and reproducibility in future studies. We also emphasize the importance of public data sharing and multicenter prospective validation to enhance generalizability.

Underrepresentation of EUS and Multimodal AI: Only 8% of studies utilized EUS, despite its clinical utility for worrisome features. The lack of NLP/LLM applications is notable. Suggestion: Address this gap in the discussion, proposing EUS-based AI and multimodal (imaging + clinical data) approaches as future directions. **[R5.3]**

Thank you for this comment. We have addressed this point in the revised Discussion section, which was also expanded in response to Reviewer 3 [R3.8]. We now highlight the underutilization of EUS and the potential of combining imaging and textual data for future AI models. Specifically, we discuss applications of vision-language models in structured report generation, multimodal risk stratification, and guideline-aligned decision support.

Clinical Translation: Only one study (Cao et al., 2023) reached prospective clinical evaluation. The absence of FDA-approved tools contrasts with AI advancements in other cancers (e.g., breast). Suggestion: Expand the discussion on barriers to translation (e.g., regulatory hurdles, clinician trust) and potential solutions (e.g., real-world validation frameworks). **[R5.4]**

Thank you. We have revised the Discussion section to address this point in detail. Specifically, we now discuss regulatory, technical, and clinical barriers to AI translation in IPMN care, and cite the recent FDA Breakthrough Device designation for PANDA [10] as an example of progress in pancreatic cancer AI tools. These additions were also made in response to a similar comment by Reviewer #3 [R3.3].

Minor Edits

Clarify Metrics: In Figure 3C, specify if reported metrics are median/mean values and their ranges. **[R5.5]**

Thank you for this important suggestion. We have specified in Supplementary Table 2 whether metrics are reported as mean or median, with confidence intervals where available. As Figure 3C provides a visual summary, no changes to the figure legend were made.

Funding Statement: The NIH grant is appropriately disclosed but clarify if funders influenced study design/outcomes. **[R5.6]**

Thank you. We would like to confirm that the statement "The content is solely the responsibility of the authors and does not necessarily represent the official views of the National Institutes of Health" was included to indicate that the funders had no role in the study design, data collection, analysis, interpretation, or decision to publish. This language follows NIH guidance on disclosures.

References: Ensure all citations (e.g., PROBAST, QUADAS) are consistently formatted. **[R5.7]**

Thank you for your attention to detail. We have ensured that all references are formatted consistently and per PLOS guidelines.

References for the Point-by-Point Response

1. Khalaf N, Liu Y, Kramer JR, El-Serag HB, Kanwal F, Singh H. Defining and understanding diagnostic delays among pancreatic cancer patients: A retrospective cohort study. Clin Gastroenterol Hepatol. 2025;23: 179–181.e3. doi:10.1016/j.cgh.2024.07.006
2. McGuigan A, Kelly P, Turkington RC, Jones C, Coleman HG, McCain RS. Pancreatic cancer: A review of clinical diagnosis, epidemiology, treatment and outcomes. World J Gastroenterol. 2018;24: 4846–4861. doi:10.3748/wjg.v24.i43.4846
3. Wolff RF, Moons KGM, Riley RD, Whiting PF, Westwood M, Collins GS, et al. PROBAST: A Tool to Assess the Risk of Bias and Applicability of Prediction Model Studies. Ann Intern Med. 2019;170: 51–58. doi:10.7326/M18-1376
4. Whiting P, Rutjes AWS, Reitsma JB, Bossuyt PMM, Kleijnen J. The development of QUADAS: a tool for the quality assessment of studies of diagnostic accuracy included in systematic reviews. BMC Med Res Methodol. 2003;3: 25. doi:10.1186/1471-2288-3-25
5. Hickman SE, Woitek R, Le EPV, Im YR, Mouritsen Luxhøj C, Aviles-Rivero AI, et al. Machine learning for workflow applications in screening mammography: Systematic review and meta-analysis. Radiology. 2022;302: 88–104. doi:10.1148/radiol.2021210391
6. Wynants L, Van Calster B, Collins GS, Riley RD, Heinze G, Schuit E, et al. Prediction models for diagnosis and prognosis of covid-19: systematic review and critical appraisal. BMJ. 2020;369: m1328. doi:10.1136/bmj.m1328
7. Jayakumar S, Sounderajah V, Normahani P, Harling L, Markar SR, Ashrafian H, et al. Quality assessment standards in artificial intelligence diagnostic accuracy systematic reviews: a meta-research study. NPJ Digit Med. 2022;5: 11. doi:10.1038/s41746-021-00544-y
8. Cohen JF, McInnes MDF. Deep learning algorithms to detect fractures: Systematic review shows promising results but many limitations. Radiology. 2022;304: 63–64. doi:10.1148/radiol.212966
9. Kolbinger FR, Veldhuizen GP, Zhu J, Truhn D, Kather JN. Reporting guidelines in medical artificial intelligence: a systematic review and meta-analysis. Commun Med (Lond). 2024;4: 71. doi:10.1038/s43856-024-00492-0
10. Cao K, Xia Y, Yao J, Han X, Lambert L, Zhang T, et al. Large-scale pancreatic cancer detection via non-contrast CT and deep learning. Nat Med. 2023;29: 3033–3043. doi:10.1038/s41591-023-02640-w
11. Yip-Schneider MT, Qadir MI, Max Schmidt C, Baril J, Rauniyar S, Lu J, et al. Artificial Intelligence in pancreatic intraductal papillary mucinous neoplasm imaging: A systematic review and meta-analysis. OSF Registries; 2024. doi:10.17605/OSF.IO/NVGXB
12. Muhammad Ibtsaam Qadir, Jackson Baril, Michele T. Yip-Schneider, Duane Schonlau, Thi Thanh Thoa Tran, C. Max Schmidt, Fiona Kolbinger. Artificial Intelligence in Pancreatic Intraductal Papillary Mucinous Neoplasm Imaging: A Systematic Review and Meta-Analysis. PROSPERO 2024 Available from https://www.crd.york.ac.uk/PROSPERO/view/CRD42024575960
