# Supplementary material for: Artificial intelligence in pancreatic intraductal papillary mucinous neoplasm imaging: A systematic review
Source: PLOS Digit Health. 2025 Jul 23;4(7):e0000920. doi: 10.1371/journal.pdig.0000920 (PMC12286379; doi:10.1371/journal.pdig.0000920)
Supplement: Attachment — Submitted filename: PDIG-D-24-00557_reviewer.docx [file pdig.0000920.s008.docx]

**Paper ID:** PDIG-D-24-00557

**Paper title:** “*Artificial Intelligence in Pancreatic Intraductal Papillary Mucinous Neoplasm Imaging: A Systematic Review****”*.**

**General Comments**

This systematic review provides a timely and comprehensive synthesis of the current state of AI applications in IPMN imaging. The manuscript is well-structured, adhering to PRISMA and AMSTAR guidelines, and addresses a clinically relevant gap in the specificity of IPMN management. The authors effectively highlight the potential of AI to mitigate overtreatment and improve diagnostic accuracy. However, several methodological and translational limitations warrant discussion to strengthen the manuscript.

**Recommendations for Authors**

**1. Strengths**

- **Scope and Rigor**

The review encompasses 25 studies (2018–2023), offering a robust analysis of AI methodologies (e.g., CNNs, radiomics) and imaging modalities (CT, MRI, EUS). The stratification by prediction targets (detection, segmentation, diagnosis, risk stratification) is particularly insightful.

- **Clinical Relevance**

The discussion on the limitations of Fukuoka/Kyoto guidelines and the role of AI in enhancing specificity is compelling. The emphasis on multicenter collaboration and multimodal data integration aligns with unmet clinical needs.

- **Transparency**

Prospective registration (OSF, PROSPERO) and detailed risk-of-bias assessment (PROBAST) enhance reproducibility.

**2. Limitations and Suggestions for Improvement**

- **Heterogeneity of Studies**
- The wide variability in cohort sizes (e.g., 27 to 9,939 patients), imaging modalities, and performance metrics (e.g., AUC: 0.624–0.996) complicates cross-study comparisons. A meta-analysis might be unfeasible, but a qualitative synthesis of high-performing models (e.g., nnUNet for segmentation) could be expanded.
- Suggestion: Include a table summarizing top-performing models per use case with key metrics (e.g., sensitivity, specificity) and dataset characteristics.
- **Data Availability and Bias**
- Most studies used single-centre, retrospective datasets with unclear selection criteria, raising concerns about generalizability. Only 32% of studies underwent external validation.
- Suggestion to discuss potential biases (e.g., selection, spectrum bias) and advocate for standardized reporting (e.g., STARD-AI, TRIPOD-AI) in future studies.
- **Underrepresentation of EUS and Multimodal AI**
- Only 8% of studies utilized EUS, despite its clinical utility for worrisome features. The lack of NLP/LLM applications is notable.
- Suggestion: Address this gap in the discussion, proposing EUS-based AI and multimodal (imaging + clinical data) approaches as future directions.
- **Clinical Translation**
- Only one study (Cao et al., 2023) reached prospective clinical evaluation. The absence of FDA-approved tools contrasts with AI advancements in other cancers (e.g., breast).
- Suggestion: Expand the discussion on barriers to translation (e.g., regulatory hurdles, clinician trust) and potential solutions (e.g., real-world validation frameworks).

**3. Minor Edits**

- Clarify Metrics: In Figure 3C, specify if reported metrics are median/mean values and their ranges.
- Funding Statement: The NIH grant is appropriately disclosed but clarify if funders influenced study design/outcomes.
- References: Ensure all citations (e.g., PROBAST, QUADAS) are consistently formatted.

**Recommendations for the Editor**

This review effectively maps the evolving landscape of AI in IPMN imaging, underscoring its promise and challenges. By addressing the above points, the authors can further enhance the manuscript’s impact as a reference for researchers and clinicians. The call for multicentre, multimodal datasets and translational efforts is both timely and necessary.

**Decision**

Recommendation Accept with minor revisions.
